# Validating Poly(3,4-ethylene dioxythiophene) Polystyrene Sulfonate-Based Textile Electroencephalography Electrodes by a Textile-Based Head Phantom

**DOI:** 10.3390/polym13213629

**Published:** 2021-10-21

**Authors:** Granch Berhe Tseghai, Benny Malengier, Kinde Anlay Fante, Lieva Van Langenhove

**Affiliations:** 1 Department of Materials, Textiles and Chemical Engineering, Ghent University, 9000 Gent, Belgium; Benny.Malengier@UGent.be (B.M.); Lieva.VanLangenhove@UGent.be (L.V.L.); 2 Jimma Institute of Technology, Jimma University, Jimma P.O. Box 378, Ethiopia; kinde.anlay@ju.edu.et

**Keywords:** textile electrode, e-textile, conductive polymer, EEG, head phantom, PEDOT:PSS

## Abstract

It is important to go through a validation process when developing new electroencephalography (EEG) electrodes, but it is impossible to keep the human mind constant, making the process difficult. It is also very difficult to identify noise and signals as the input signal is unknown. In this work, we have validated textile-based EEG electrodes constructed from a poly(3,4-ethylene dioxythiophene) polystyrene sulfonate:/polydimethylsiloxane coated cotton fabric using a textile-based head phantom. The performance of the textile-based electrode has also been compared against a commercial dry electrode. The textile electrodes collected a signal to a smaller skin-to-electrode impedance (−18.9%) and a higher signal-to-noise ratio (+3.45%) than Ag/AgCl dry electrodes. From an EEGLAB, it was observed that the inter-trial coherence and event-related spectral perturbation graphs of the textile-based electrodes were identical to the Ag/AgCl electrodes. Thus, these textile-based electrodes can be a potential alternative to monitor brain activity.

## 1. Introduction

**Figure 1:**
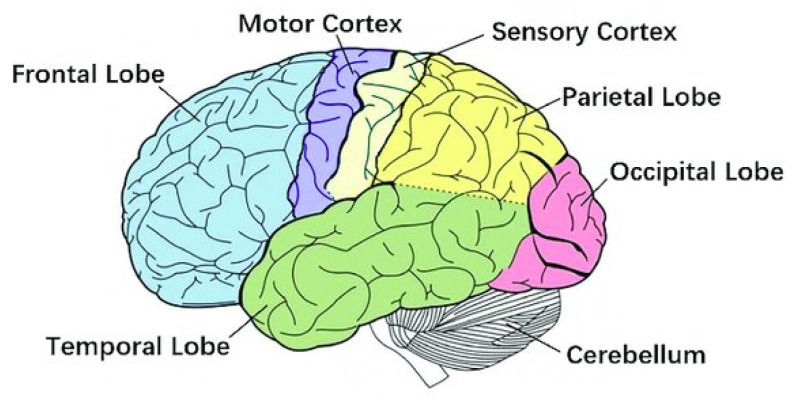
The human brain [3], under CC by 4.0.

The brain is the largest and most complex organ in the human body, consisting of more than 100 billion nerves [1]; it is considered as the central organ of the human nervous system and, together with the spinal cord, forms the central nervous system [2]. The condition of the brain can be diagnosed through an electroencephalogram (EEG) measurement, where the current flow within its regions (Figure 1) is determined and displayed as a wave. Each person’s brain wave patterns are unique, which makes it possible to distinguish between people only based on their typical brain activity.

The first human EEG was reported in 1929 by Hans Berger [4]. This measurement technique is often employed in the diagnosis of neurological disorders [5], the intensity of anesthesia [6], encephalopathies [7], brain death [8], and so on. Novel applications such as brain–computer interface [9], robotic rehabilitation of patients [10], and the investigation of brain development [11] increased the interest in EEG measurements.

EEG waveforms are classified based on their frequency and denoted with Greek numerals based on their spectrum as delta (0.5 to 4 Hz), theta (4 to 7 Hz), alpha (8 to 12 Hz), sigma (12 to 16 Hz), beta (13 to 30 Hz), and gamma (30 to 100 Hz) [12]. Delta waves, which have the lowest frequency and highest amplitude, reflect the activity of the gray matter in the brain. They occur in all stages of sleep, but are uncommon in awake adults. Theta waves usually occur in the parietal and temporal regions in children, and in some adults, during times of frustration and emotional stress. Alpha waves originate on both sides of the head and can collect in the occipital and parietal regions. They usually occur on waking, but relax with closed eyes and disappear completely during sleep. These waves represent the white matter of the brain and connect conscious and subconscious states. Sigma waves appear predominantly in the frontal-central head regions during sleep and are called sleep spindles. They quickly occur, similar to the shape of an “eye” in that they rapidly increase in amplitude and then quickly decay. Beta waves can be detected on both sides of the frontal and parietal lobes during active brain states such as speech, problem-solving, judgment, and decision making. Gamma waves are waves that occur during hyperactive wakefulness with the integration of sensory inputs and indicate the connection of feelings with memory activity.

**Figure 2:**
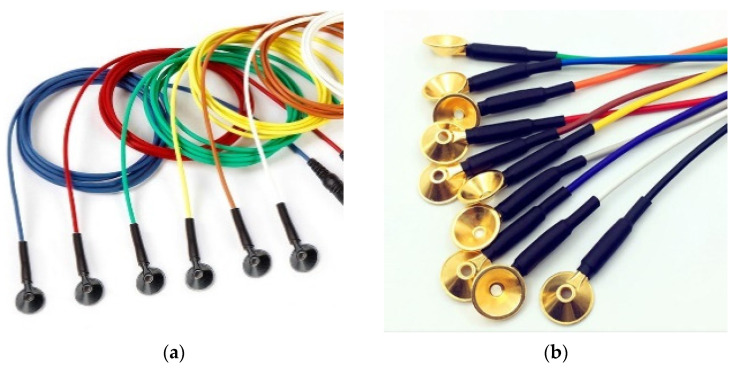
EEG cup wet electrodes: (**a**) Ag/AgCl and (**b**) Au plated.

Existing EEG measurements are done in hospitals and clinics using wet electrodes (e.g., Ag/AgCl electrodes), which have many drawbacks: abrasive lesions during skin preparation, allergic reactions from the conductive gel, and artifacts caused by moisture change. Highly skilled professionals are required and the patients need to stay for a long time at the diagnosis location. Furthermore, skin preparation and gel application take time when a high number of electrodes are necessary. Wet electrodes are single-use and thrown away, and thus increase the cost per diagnosis and produce a large amount of waste. Some examples of standard wet electrodes are shown in Figure 2.

**Figure 3:**
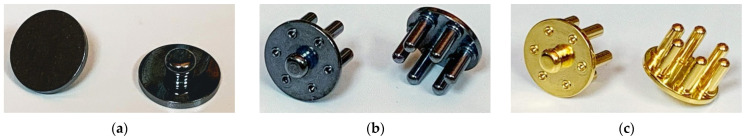
Reusable dry EEG electrodes: (**a**) flat dry Ag/AgCl electrode; (**b**) spike dry Ag/AgCl electrode; and (**c**) spike dry gold plated electrode.

The aforementioned problems associated with wet electrodes caused a demand for more comfortable and user-friendly electrodes that has, in turn, led to the development of an increasing number of dry electrodes capable of overcoming the limitations of wet electrodes. However, the dry electrodes have a rigid structure and are relatively heavyweight, making them not suitable for long-term monitoring and wearable applications. Some examples of commercial dry electrodes are shown in Figure 3.

**Figure 4:**
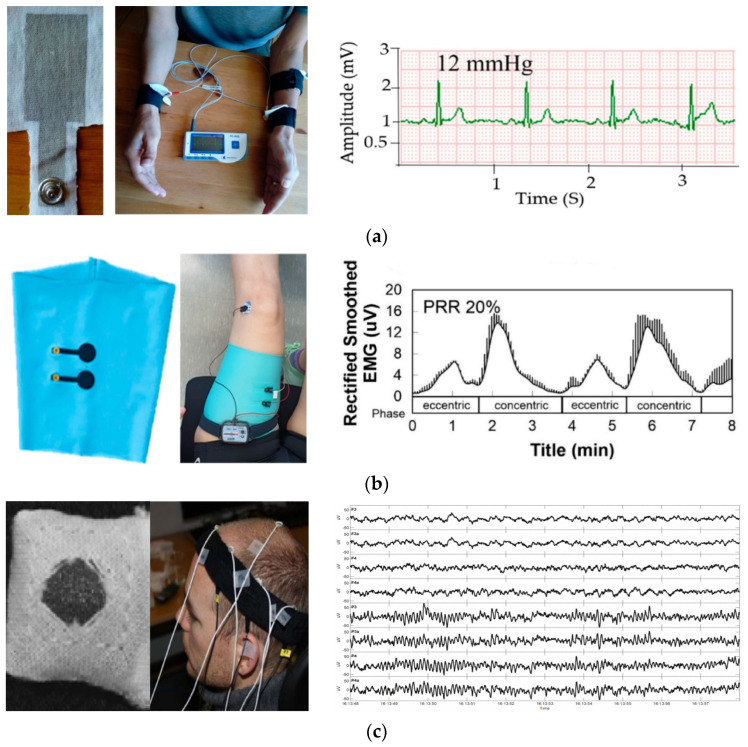
Textile-based bio-potential sensors: (**a**) silver-printed cotton electrocardiography electrode [13], under CC by 4.0; (**b**) silver and carbon paste conductive sheet electromyography electrodes laminated polyester/spandex sleeve [15], under CC by 4.0; (**c**) 15% nylon, 30% silver-plated conductive fibers, 20% Spandex, and 35% polypropylene knitted fabric electroencephalography electrode [16], under CC by 4.0.

On the other hand, the combination of textile material with electronics led to a new class of large-area, flexible, conformable, and interactive smart textiles. New value-added textile products led to significant results in wearable multifunctional smart textiles, enabling several applications in healthcare, protection, fashion, military, and so on. Similarly, the use of textile electrodes for biopotential sensing applications has been booming and demands the attention of textile, electronic, and medical expertise owing to their flexibility and low weight, which are advantages over existing commercial electrodes. For instance, textile-based electrocardiography electrodes [13,14], electromyography [15], and EEG [16,17] have been reported recently. Some typical examples of textile electrodes reported for biopotential sensing are shown in Figure 4.

**Figure 5:**
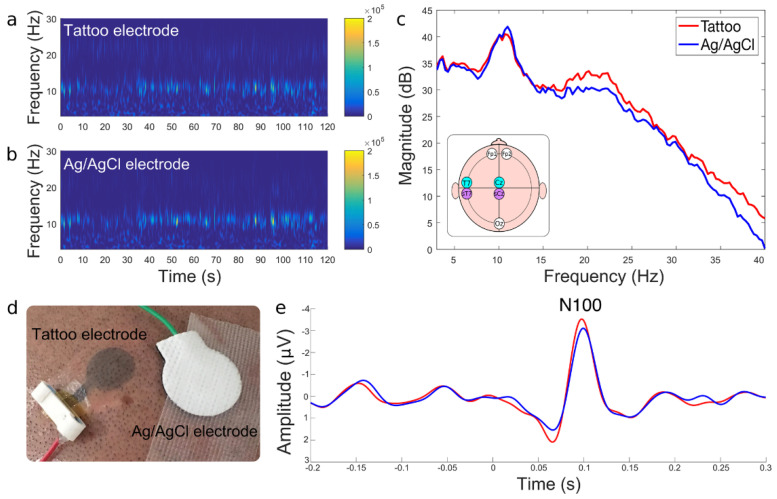
Time–frequency plot of alpha wave recordings, with visible 10 Hz activity obtained with (**a**) tattoo and (**b**) AgAgCl electrodes; (**c**) superimposed power spectral density (dB) during alpha wave recordings from the textile tattoo electrodes (TTEs)—in red, and Ag/AgCl electrodes—in blue. The insert at the bottom left shows the placement of the electrodes with used derivation (Tz–Cz, in light blue for the TTEs, and sT7-sCz for the Ag/AgCl electrodes in light violet); (**d**) picture of two electrodes in Cz position on the head of the participant; (**e**) auditory evoked potential recorded with both TTEs (red) and Ag/AgCl electrodes (blue), with N100—an auditory evoked potential component [20]. Under CC by 4.0.

From a textile perspective, the aim is to produce the entire component, like sensors, actuators, transmission lines, and so on, from 100% textile material [18]. However, the use of metallic particles like silver, copper, and gold does not support this target as the texture of the textile is often compromised. Metallic particles impart stiffness to the fabric and almost cause them to lose their textile characteristics. In addition, the formulation of metallic inks is a complex process requiring a lot of additives and specialized processes, and is thus expensive. Hence, the use of conductive polymers for biopotential sensing electrodes like EEG could be a problem-solving alternative owing to their highly effective contact areas with human skin, biocompatibility, high electrical conductivity, and inherent mechanical flexibility [19]. Among the conductive polymers, poly(3,4-ethylene dioxythiophene) polystyrene sulfonate (PEDOT/PSS) has gained attention and has been already reported in different formats. For instance, Laura et al. [20] have developed a PEDOT/PSS tattoo EEG electrode, as shown in Figure 5.

Though the EEG tattoo approach is novel and seemingly promising, it only remains until washing and is not reusable in the case of temporary tattoos and fades in case of permanent tattoos owing to skin development. Moreover, tattoos may cause an allergic reaction in humans, resulting in a rash that is typically red, bumpy, or itchy. These symptoms may appear in the days following the initial tattooing or months or years later. Thus, reusable textile-based electrodes are still a primal choice as long as the concern is long-term monitoring and from the economic point of view.

Whatever the nature of the electrodes, it has to be validated before being employed in clinical practices. For instance, PEDOT/PSS-based [14] and silver-based [13] electrocardiography (ECG) electrodes have been reported to measure heart activity, but a scientific validation was not performed as part of that research as ECG signals were different from person to person and even for the same person over time. Similarly, we have also used poly(3,4-ethylenedioxythiophene) polystyrene sulfonate/polydimethylsiloxane (PEDOT/PSS/PDMS) electrodes to measure brain activity [17,21], and noticed that it is much more variable, with changes occurring over seconds. Thus, in this work, we focused on the validation of PEDOT/PSS/PDMS-based EEG electrodes. During the validation of EEG electrodes, the brain waves should be collected directly from a human. However, maintaining the human mind stable is not practically possible. It is also difficult to exactly identify the extent of noise and signals as the original currents flowing in the brain are unknown.

As a solution, researchers came up with anatomically realistic head phantoms mimicking the brain such as digital phantoms [22] and ballistic gelatin [23,24,25,26,27]. On the motivation of overcoming the electromagnetic interference noise generated by the power lines, and high power electronic equipment in digital phantoms [28] and the short life span [29] and too heavyweight of ballistic gelatins, we reported a long-lasting and anatomically realistic textile-based head phantom that gives low skin-to-electrode impedance and better signal-to-noise ratio than ballistic gelatin [12]. Therefore, in this work, this head phantom was used to validate the PEDOT/PSS/PDMS-based EEG electrodes.

## 2. Experiment

### 2.1. Conductive Fabric Development

**Figure 6:**
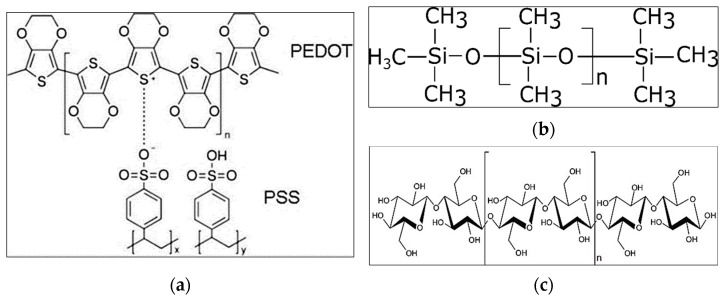
(**a**) The chemical formula of PEDOT/PSS [36], under CC by 4.0; (**b**) the chemical structure of PDMS; (**c**) the chemical structure of a polymeric unit of cotton, cellulose, which is a linear polymer made up of β-d-glucopyranose units covalently linked with (1–4) glycosidic bonds [37], under CC by 3.0.

The development of the conductive fabric followed previous work, Tseghai et al. [30]. Knitted cotton fabric is used as a textile substrate owing to its wearing comfort and is adequately available. A high-conductivity grade PEDOT/PSS PH1000 Clevious conductive polymer obtained from (Ossila Ltd., Belfast, UK) and a biocompatible poly(dimethylsiloxane (PDMS) elastomer obtained from (Polyscience, Inc., Hirschberg, Germany) were used to produce a conductive polymer composite. PEDOT/PSS was selected because of its acceptable electrical conductivity and flexibility [31] and its biocompatibility [32]. PDMS was chosen because of its biocompatibility and extensibility [33]. In addition, PDMS has several properties making it favorable such as low cost [34] and transparent (240 nm–1100 nm range) [35] products. 1,2,3,4-Butanetetracarboxylic acid (BTCA) obtained from (Sigma-Aldrich, Inc., Darmstadt, Germany) was also used as a fixing agent to improve wash fastness. The chemical structures of PEDOT/PSS, PDMS, and cellulose, the polymeric units of cotton, are shown in Figure 6.

**Figure 7:**
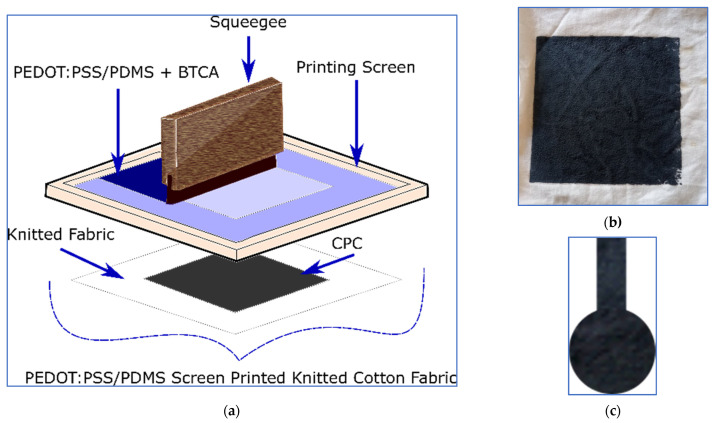
(**a**) The schematic illustration of flat screen printing [21], under CC by 4.0; (**b**) PEDOT/PSS/PDMS-printed knitted cotton fabric; and (**c**) actual EEG textile electrode.

Similar to Tseghai et al. [21], a 1:4 proportion of PDMS to PEDOT/PSS was mixed using a stirring rod until a homogenous PEDOT/PSS–PDMS paste was obtained. Then, BTCA, 10% of the weight of paste, was added to the recipe. The PEDOT/PSS/PDMS paste was next screen printed to knitted cotton fabric and, finally, the printed fabric was dried at 70 °C for 10 min and cured at 150 °C for 3 min. The schematic illustration of the overall printing process and the actual PEDOT/PSS/PDMS-printed conductive knitted cotton fabric are shown in Figure 7a,b, respectively.

### 2.2. Textile Electrode Design and Construction

The textile electrode design and construction were also according to our previous work, Tseghai et al. [21], where a 67.23 Ω/sq electrically resistive conductive fabric was employed. The fabric behaves as a twill 1/4 cotton fabric or a twill 2/1 kermel/viscose of the same approximate gram per square reported by Musa et al. [38]. The mechanical stability of the work has also been studied in-depth in that article; the textile electrodes were found to be robust to 60 bending, 15 washing cycles, and 5 multiple uses, providing reliable EEG signals [21]. Five replicas of electrodes with 2 cm diameter, as shown in Figure 7c, were constructed to examine the repeatability and reproducibility of signal quality. In addition, the signal qualities were compared against a dry Ag/AgCl electrode.

### 2.3. Head Phantom Construction

**Figure 8:**
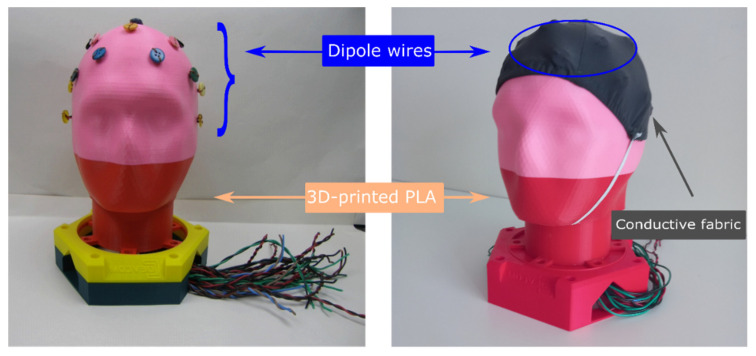
Textile-based head phantom [12], under CC by 4.0.

A double-sided conductive (18:7) nylon/spandex elastomeric fabric (from MANDU, Helsinki, Finland) of 1 × 10^4^ to 1 × 10^7^ Ohm/sq was placed over a real 3D printed polylactic acid (PLA) skull to construct a textile head phantom. Twenty bipolar cables were routed under the conductive tissue, as shown in Figure 2a. The skull, base-ring, and inner post were made from PLA using a 3D printer at Ingegno Maker Space. The textile-based head phantom used for validating the textile-based electrodes is shown in Figure 8.

### 2.4. Synthetic Sine Wave Generation

**Figure 9:**
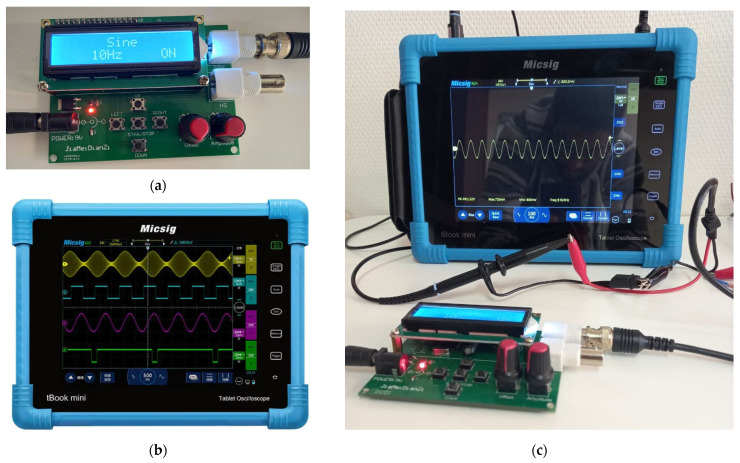
Synthetic sine wave generation: (**a**) function generator printing; (**b**) digital oscilloscope; and (**c**) synthetic sine wave generated by the function generator on a digital oscilloscope.

A synthetic sine wave was generated using a portable digital oscilloscope Micsig TO1104 (peak to peak voltage 360 mV, maximum voltage 168 mV, minimum voltage −192 mV, frequency 9.925 Hz, duration 50 ms), and then injected into the textile phantom as shown in Figure 9. For mimic events, the parameters of the EEG signal were set in the alpha wavelength range and the amplitude was changed to simulate a neural event. This synthetic sine wave was used for both the phantom-to-electrode impedance and EEG measurements through an active textile electrode connected to an OpenBCI board.

### 2.5. Phantom-to-Electrode Impedance Measurement

The electrical impedance at the skin–electrode interface influences the EEG signal quality [39,40], thus designing a dry electrode with a low skin-contact impedance improves EEG performance. In this work, the term phantom-to-electrode impedance is used as the testing was performed with a head phantom in the EEG alpha band power, not a human.

The impedance of the phantom-to-electrode was measured using a three-electrode configuration (reference electrode, counter electrode, and active electrode) as well as a Cyton Biosensing board (OpenBCI) and textile electrode to study the difference between them. The system was adopted from OpenBCI and suggested measuring the skin-to-electrode impedance measurement as the OpenBCI Cython board has an ADS1299 for impedance measurement. The ADS1299 has a “Lead Off Detection” feature that allows it to measure impedance by injecting a known current into each electrode. An Ag/AgCl dry electrode was used for comparison under the same testing conditions.

**Figure 10:**
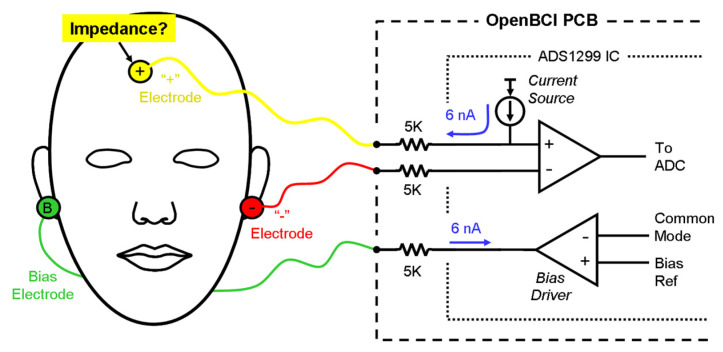
Skin-to-electrode impedance measurement using an OpenBCI system that possesses an ADS1299 to measure impedance.

The ADS1299 [41] forces a 6 nA current into the electrode line regardless of the resistance or impedance between the current source and the ground. As a result, a 6 nA current was flown through the electrode to the ground during this test. A 5 kΩ resistor is built into the OpenBCI board in series with each electrode, as shown in Figure 10. In addition, the average voltages measured during the test are expressed as root mean square voltages (Vrms). Therefore, the phantom-to-electrode impedance was calculated using Equation (1).

(1) $$\mathrm{Actual}\;\mathrm{Average}\;\mathrm{Impedance}\;\left(\Omega \right)\;=\frac{\mathrm{Vrms}\times 2\sqrt{2}\;\left(V\right)}{\pi \times \mathrm{Current}\;\left(A\right)}-\mathrm{Load}\;\mathrm{Resistance}\;\left(\Omega \right)$$

where the current and the load resistance are 6 nA and 5 kΩ, respectively.

### 2.6. EEG Measurement and Analysis

**Figure 11:**
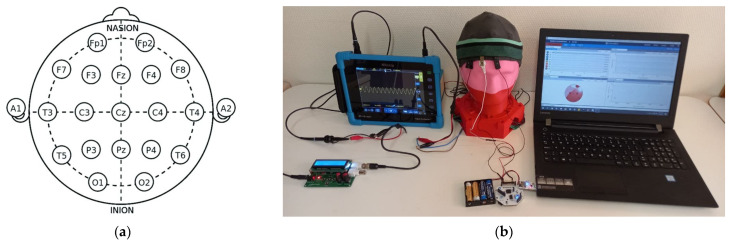
(**a**) The 10–20 international system of EEG electrode placement [42], under CC by 4.0; (**b**) synthetic scheme 2. The initial peak-to-peak voltage signal is the synthetic peak-to-peak voltage injected from the digital oscilloscope to the head phantom. Thus, the actual peak-to-peak voltage signals are the peak-to-peak voltage collected from the head phantom and the peak-to-peak voltage noise is the difference between the injected and collected back peak-to-peak voltage signal.

The international federation’s 10–20 EEG placement (Figure 11a), i.e., the most commonly used system for mounting electrodes for clinical EEG monitoring, was used to place five electrodes, three active (FP_1_, FP_2_, and F_z_) on the head and two references (A_1_ and A_2_) on the earlobe. The 10–20 system of electrode placement is a method for describing where scalp electrodes should be placed [42]. These scalp electrodes are used to record the electroencephalogram (EEG) with an electroencephalograph machine. To hold the electrode in the required positions, a tight-fitting headband made of elastic bandage was used. The EEG waveforms were recorded with eight channels at a sampling frequency of 250 Hz for 300 s via an OpenBCI board at 60 Hz notch and 1–50 Hz bandpass filter; the EEG measurement setup is shown in Figure 11b. Each channel measures the difference between one electrode and a reference electrode as the referential electrode installation was followed. In this type of installation, the reduction in disturbances and noise is common for all the electrodes.

(2) $$\mathrm{SNR}\;\left(\mathrm{dB}\right)\;=10\log\left(\frac{\mathrm{Peak}\;\mathrm{to}\;\mathrm{Peak}\;\mathrm{Voltage}\;\mathrm{Signal}}{\mathrm{Peak}\;\mathrm{to}\;\mathrm{Peak}\;\mathrm{Voltage}\;\mathrm{Noise}}\right)$$

Finally, an EEGLAB software [41] was used to perform data treatment and statistics offline. A 250 Hz low pass filter, 512 Hz resampling, and a 0.5 Hz high pass filter were used in the beginning. ERP was obtained for the time domain analysis by averaging baseline-corrected epochs taken from 0.5 to 2.5 s after the target apparition event. From the initial 1960 epochs (5% discarded), a total of 1862 epochs remained after artifact rejection. Meanwhile, intertrial coherence (ITC) was obtained for wave cycles from 3 to 0.5, epoch time limit from 0 to 1960, and frequency limit from 0.5 to 250 Hz. ITC was analyzed via EEGLAB software that is treated as in Equation (3) according to spectral and coherence estimates on EEG recordings [21].

(3) $$\mathrm{ITC}\;\left(f,t\right)\;=\frac{1}{n}\overset{n}{\underset{k=1}{\sum }}\frac{F_{k}\;\left(f,\;t\right)}{|F_{k}\;\left(f,\;t\right)|\;}$$

where, F, *t*, and n denote frequency, time, and numbers of data, respectively.

### 2.7. On-Body EEG Measurement

**Figure 12:**
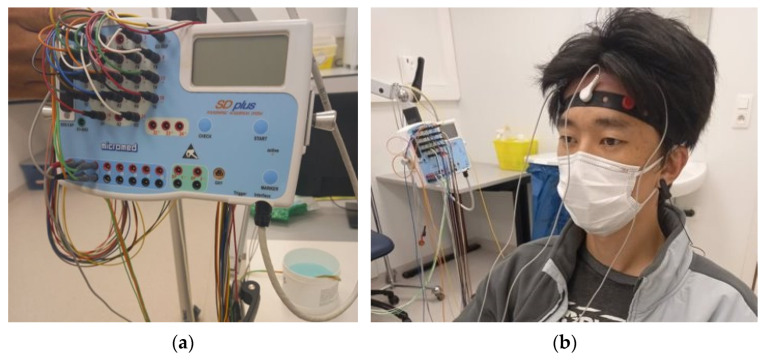
Electroencephalography (EEG) measurement: (**a**) Brain Quick EEG Clinical line and (**b**) photographic image of actual EEG measurement.

As a proof of concept, on-body EEG measurement was performed with a volunteer subject at Ghent University Hospital, Neurology Department. All the study were approved by Ethical Clearnce Committee of EiTEX (04-11-2020). Brain QUICk EEG Clinical Line (Figure 12a) was used to conduct the EEG measurement using three active electrodes and two more reference electrodes. The active electrodes were placed on Fp1, Fpz, and Fp2 head positions, and the reference electrodes were placed in the earlobe, as shown in Figure 12b. An on-body EEG measurement was also performed with Ag/AgCl dry electrodes for comparison.

## 3. Results

### 3.1. Phantom-to-Electrode Impedance

Table 1 shows the average voltage and respective raw and actual phantom-to-electrode impedance of the textile and dry Ag/AgCl electrodes. The phantom-to-electrode impedance of the textile-based electrode is significantly lower than the commercial dry electrode with an f-ratio and *p*-value of 12.75 and 0.003, respectively, at a 95% confidence interval based on one-way ANOVA. On top of that, the impedance is less than half of the required value to detect an EEG signal, i.e., 5000 Ω [43]. Therefore, the textile-based electrode can be potentially used to acquire EEG signals in a wearable application. The actual values of the phantom–electrode impedance could vary owing to other factors.

### 3.2. EEG Signal Analysis

#### 3.2.1. Amplitude and Frequency

**Figure 13:**
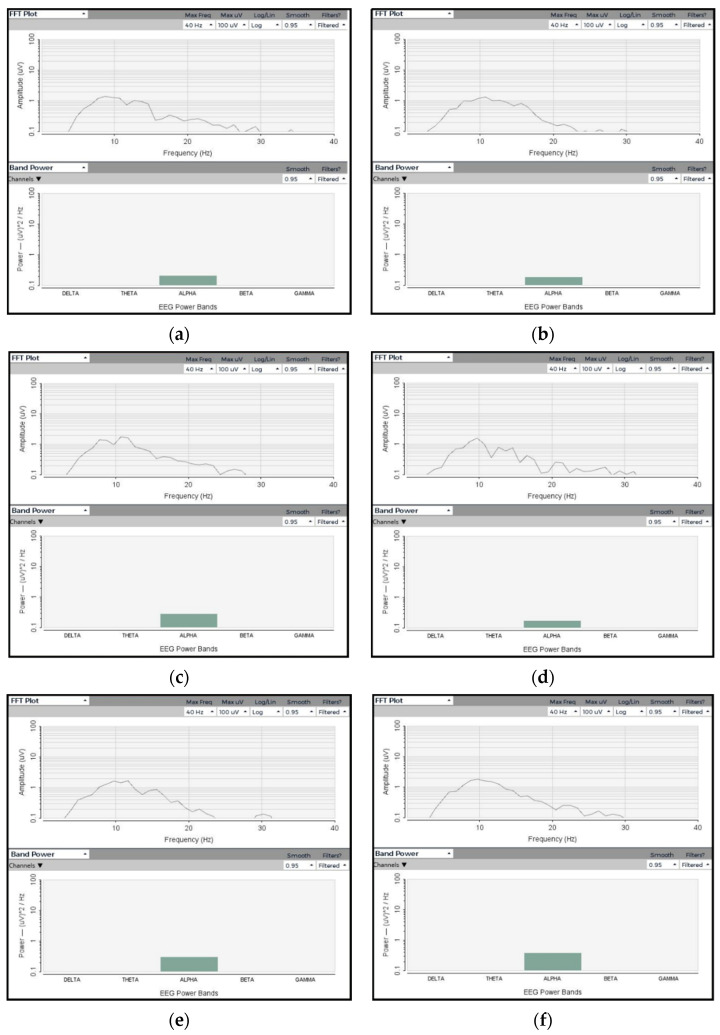
EEG signals from OpenBCI board on textile-based head phantom: (**a**) Ag/AgCl; (**b**) textile electrode 1; (**c**) textile electrode 2; (**d**) textile electrode 3; (**e**) textile electrode 4; and (**f**) textile electrode 5.

The EEG signals in Figure 13 indicate that the textile electrodes are capable of acquiring EEG signal quality equivalent to Ag/AgCl electrodes. All five textile electrodes have collected the wave predominantly at the alpha band as in the injected sine wave. The amplitudes and band powers collected by the five textile electrodes are identical and equivalent to the Ag/AgCl electrode. Therefore, the textile electrodes give reliable EEG signal amplitude and band power that are important for long-term monitoring of brain activity.

#### 3.2.2. SNR Analysis

External noise or artifacts in EEG are defined as any signal picked up by the sensors but not generated by the brain; for this case, by the phantom. Noise or artifacts in EEG data can come from a variety of sources. Anything that uses electricity emits an electromagnetic field, which your measuring equipment may be able to detect.

The signal-to-noise ratio (SNR) is the ratio of desired signal power to undesired information or background noise power, which is often expressed in decibels [44]. SNR is a scientific and engineering measurement that compares the level of the desired signal to the level of background noise.

From Table 2, the average SNR of five replica has been found to be 17.378 dB ± 0.0716 (±0.41%) at a 95% confidence interval, which shows the values are not significantly different. In addition, the SNR of the textile electrodes is higher (+3.45 dB) than the Ag/AgCl electrode. Therefore, the sensing reputability of the textile-based EEG electrode is excellent. In this work, only three active electrodes were used to measure the EEG. Using more active electrodes, increasing the duration of measurement, increasing the size of the electrode, and using a clinical EEG machine would also enhance the SNR to a better level. On the other side, if the EEG measurement was performed with humans, the SNR would be affected by motion artifacts and other physiological activities. Further study on the SNR correlation of the brain and the textile head phantom would be important to find out the actual values.

#### 3.2.3. ERSP and ITC

The event-related spectral perturbation (ERSP) plots mean event-related spectral power fluctuations at each frequency and time during the epoch [45]. The ERSP quantifies the average time course of relative changes in the spontaneous EEG amplitude spectrum caused by a series of similar experimental events. Meanwhile, intertrial coherence (ITC) refers to the degree to which EEG activity in single trials is phase-locked at a given time and frequency (not phase-random with respect to the time-locking experimental event) [46]. It is a measure of oscillatory phase consistency across a group of trials for comparing phase synchronization between trials. At a given point in time, it is the circular sum of phases (length of the red arrow). It achieves a maximum of 1 for perfectly phase-aligned signals, indicating perfect intertrial coherence (i.e., the same phrase on every trial), and then drops to 0 as the phase distribution becomes more uniform, indicating no intertrial coherence at all. ERSP and ITC responses of the textile-based electrodes were identical. Moreover, the ERSP and ITC of the textile-based EEG electrodes were also similar, as shown in Figure 13. Thus, the PEDOT/PSS/PDMS-printed cotton fabric textile electrodes can be used to monitor brain activity. The textile electrode requires no conductive gel, taking advantage over wet and has flexible, light weight, and washable characteristics that makes it advantagous over commercial dry metallic electrodes too. In addition, it possesses the characteristics of normal textile materials, which make it suitable to be attached with any textile substrate and structure the way conventional textile materials are attached to each other.

**Figure 14:**
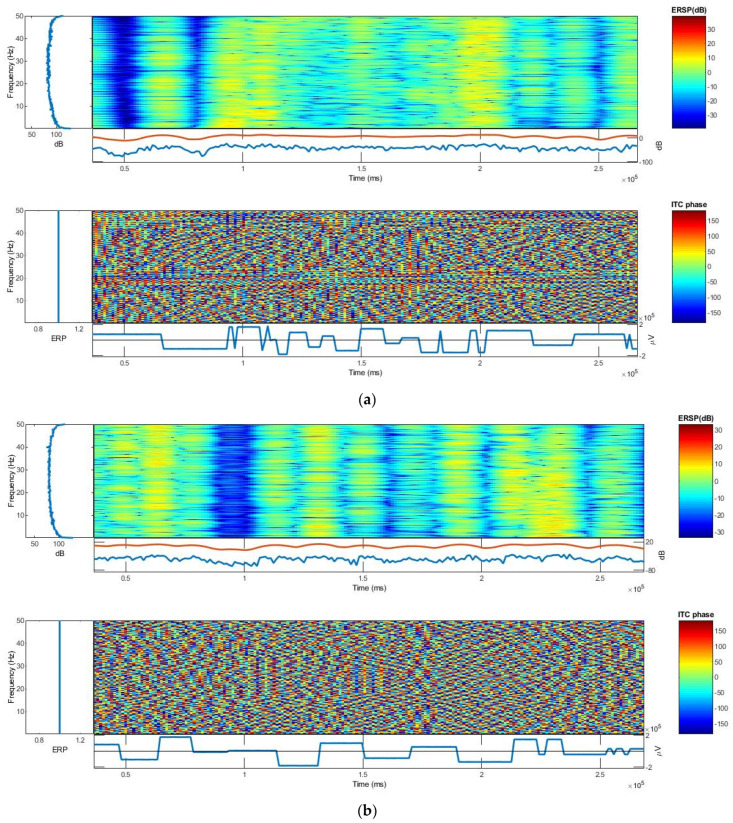
The event-related spectral perturbation (ERSP) and intertrial coherence (ITC) plots: (**a**) textile electrode and (**b**) dry Ag/AgCl electrode.

At a 99% confidence interval, the frequency and time ranges are plotted on the *y*-axis and *x*-axis, respectively, and a color scale is used, with green representing non-significant ITC and red representing significant ITC. Under each ITC plot, the averaged ERP response for phantom (in blue) is plotted. The ERP response amplitude scale for both textile and dry Ag/AgCl electrodes is somewhat similar, as shown in Figure 14. The log power spectral density for both the textile and dry Ag/AgCl electrodes was 90 dB and the distribution of spectral powers was also similar. In the range of 1 to 5 Hz frequency, generally, the ERSP and ITC responses of the textile and dry Ag/AgCl electrodes are almost identical.

### 3.3. On-Body EEG Signals

**Figure 15:**
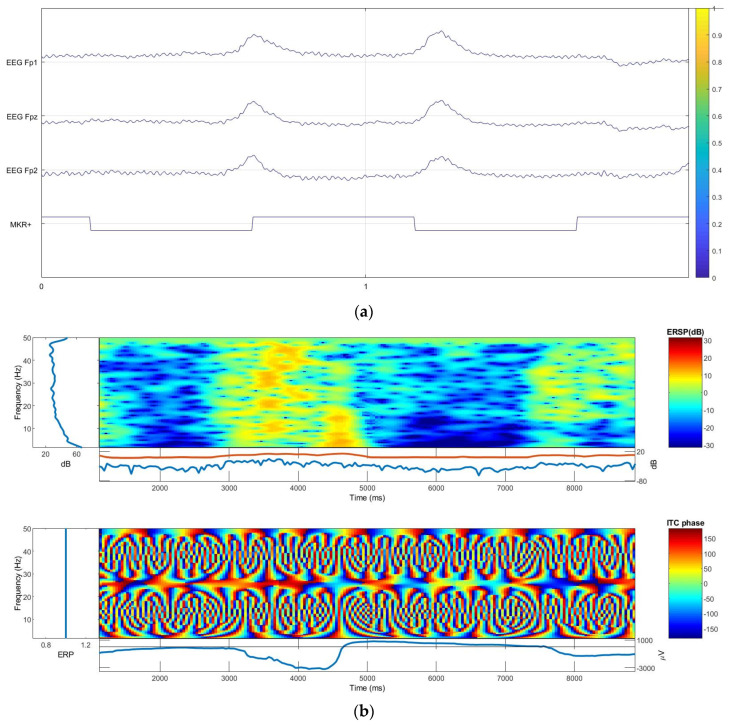

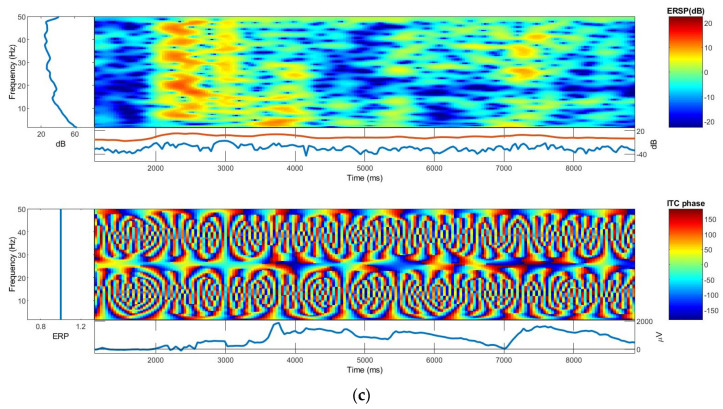
(**a**) Electroencephalography (EEG) signals from the textile electrode; (**b**,**c**) event-related spectral perturbation (ERSP) and intertrial coherence (ITC) plots: (**b**) textile electrode and (**c**) dry Ag/AgCl electrode.

From the EEG clinical line measurement, it was observed that the PEDOT/PSS/PDMS-printed textile can collect EEG signals, as shown in Figure 15a. The ITC and ERSP were also found to be identical with Ag/AgCl dry electrodes. The EEG signals, ITC, and ERSP graphs for the textile-based electrode and Ag/AgCl dry electrodes are shown in Figure 15b,c. This indicates that the PEDOT/PSS/PDMS-printed textile electrodes are promising for EEG measurement in brain activity monitoring, especially for wearable applications.

## 4. Conclusions

The need for greater comfort has prompted the development of various dry electrode formats that can overcome the limitations of wet electrodes. As a result, a dry metal EEG disc and a comb have just been introduced. However, their heavy weight and structural rigidity can render them unsuitable for wearable uses. As a solution to the above problems, a flexible conductive textile material was used to develop a washable textile electrode that detects EEG signals equivalent to dry Ag/AgCl electrodes.

In this study, we have validated a PEDOT/PSS/PDMS-based textile EEG electrode that has the properties of regular textiles and acceptable skin-to-electrode resistance. In addition, it can receive EEG signals comparable to conventional dry electrodes and shows a power spectral density similar to that of a dry Ag/AgCl dry electrode with ERSP and ITC identical plots. Thus, this validated textile electrode can be used to monitor brain activity in wearable devices. Moreover, EEG signals have also been collected from humans using textile electrodes at the clinical level.

A textile-based electrode that has a lower representation than metallic electrodes in EEG measurement and textile phantom is a completely new approach. Thus, this work also investigates the potential performance of textile electrodes and head phantoms, where emerging methods of applying conductive polymers to textile substrates may outperform the approach.

## Figures and Tables

**Table 1:** The phantom-to-electrode impedance in the EEG alpha band power.

Time (s)	Textile-Based Electrode			Dry Ag/AgCl Electrode		
	V_avg_^1^	Z_raw_^2^	Z_act_^3^	V_avg_	Z_raw_	Z_act_
30	41.18	6863.33	1863.33	43.93	7321.67	2321.67
60	43.81	7301.67	2301.67	43.98	7330	2330
90	42.66	7110	2110	44.54	7423.33	2423.33
120	42.92	7153.33	2153.33	43.87	7311.67	2311.67
150	43.13	7188.33	2188.33	42.49	7081.67	2081.67
180	42.44	7073.33	2073.33	44.12	7353.33	2353.33
210	41.33	6888.33	1888.33	43.72	7286.67	2286.67
240	42.72	7120	2120	43.63	7271.67	2271.67
Mean	41.18	6863.33	1863.33	43.79	7297.5	2297.5

^1^ Raw average voltage [µV]. ^2^ Raw average phantom-to-electrode impedance [Ω]. ^3^ Actual phantom-to-electrode impedance [Ω].

**Table 2:** Injected synthetic wave, acquired signal, and SNR of the textile-based electrodes.

	V Max (µV)	V Min (µV)	V Pk-Pk (µV)	SNR (dB)
Synthetic Wave	168,000.00	192,000.00	360,000.00	-
Ag/AgCl electrode	166,830.00	−185,800.00	352,630.00	16.8
Textile electrode 1	165,616.73	−187,825.55	353,442.27	17.32
Textile electrode 2	165,689.17	−187,867.88	353,557.04	17.39
Textile electrode 3	165,596.89	−187,795.33	353,392.21	17.28
Textile electrode 4	165,614.41	−187,967.12	353,581.53	17.41
Textile electrode 5	165,689.79	−188,001.02	353,690.81	17.49
